# FGF and EDA pathways control initiation and branching of distinct subsets of developing nasal glands

**DOI:** 10.1016/j.ydbio.2016.08.030

**Published:** 2016-11-15

**Authors:** Alison J. May, Denis Headon, David P. Rice, Alistair Noble, Abigail S. Tucker

**Affiliations:** a Department of Craniofacial Development and Stem Cell Biology, Guy's Hospital, King's College London, United Kingdom; b The Roslin Institute and Royal (Dick) School of Veterinary Studies, University of Edinburgh, Edinburgh, United Kingdom; c Orthodontics, Department of Oral and Maxillofacial Diseases, University of Helsinki, Helsinki 00014, Finland; d Orthodontics, Department of Oral and Maxillofacial Diseases, Helsinki University Hospital, Helsinki 00290, Finland; e MRC & Asthma UK Centre in Allergic Mechanisms of Asthma, King's College London, United Kingdom

**Keywords:** Submucosal gland (SMGs), FGF10, EDA, Branching morphogenesis

## Abstract

Hypertrophy, hyperplasia and altered mucus secretion from the respiratory submucosal glands (SMG) are characteristics of airway diseases such as cystic fibrosis, asthma and chronic bronchitis. More commonly, hyper-secretion of the nasal SMGs contributes to allergic rhinitis and upper airway infection. Considering the role of these glands in disease states, there is a significant dearth in understanding the molecular signals that regulate SMG development and patterning. Due to the imperative role of FGF signalling during the development of other branched structures, we investigated the role of *Fgf10* during initiation and branching morphogenesis of murine nasal SMGs. *Fgf10* is expressed in the mesenchyme around developing SMGs while expression of its receptor *Fgfr2* is seen within glandular epithelial cells. In the *Fgf10* null embryo, Steno's gland and the maxillary sinus gland were completely absent while other neighbouring nasal glands showed normal duct elongation but defective branching. Interestingly, the medial nasal glands were present in *Fgf10* homozygotes but missing in *Fgfr2b* mutants, with expression of *Fgf7* specifically expressed around these developing glands, indicating that *Fgf7* might compensate for loss of *Fgf10* in this group of glands. Intriguingly the lateral nasal glands were only mildly affected by loss of FGF signalling, while these glands were missing in *Eda* mutant mice, where the Steno's and maxillary sinus gland developed as normal. This analysis reveals that regulation of nasal gland development is complex with different subsets of glands being regulated by different signalling pathways. This analysis helps shed light on the nasal gland defects observed in patients with hypohidrotic ectodermal dysplasia (HED) (defect EDA pathway) and LADD syndrome (defect FGFR2b pathway).

## Highlights

• Murine nasal submucosal glands express *Fgf10, Fgfr2* and *EDA* during embryonic development.
• All nasal glands require FGF10 for branching morphogenesis.
• FGF10 is required for gland budding and ductal morphogenesis of the Steno's gland and the maxillary sinus glands.
• EDA signalling is essential for Lateral Nasal Gland and Medial Nasal Gland budding and ductal morphogenesis.

## 1 Introduction

Impaired mucus clearance and pulmonary obstruction are common symptoms of a number of life-threatening respiratory diseases. Mucus hyper-secretion by the submucosal glands (SMG) is an important etiological factor in asthma, chronic bronchitis and cystic fibrosis with SMG hyperplasia and mucous metaplasia common to all (Reid, 1960, Oppenheimer and Esterly, 1975, Aikawa et al., 1992). Hyper-secretion and abnormal mucociliary clearance leads to a build-up of mucus with a thick viscosity which can obstruct airways and increase bacterial lung infection, leading to premature death in severe cases (Oppenheimer and Esterly, 1975, Hoegger et al., 2014, Robinson and Bye, 2002). More commonly, altered mucus secretion of the nasal glands, particularly the sinus glands, gives rise to chronic rhinosinusitis and infection of the upper airway tract (Peña et al., 2007, Wu et al., 2011). Considering this significant involvement of SMGs in pulmonary diseases, research is lacking in the mechanisms modulating gland development and homeostasis. To understand the progression of airway disease, it is critical to elucidate the signalling factors and pathways required during SMG morphogenesis, and investigate if these mechanisms are defective in disease states.

**Fig. 1:**
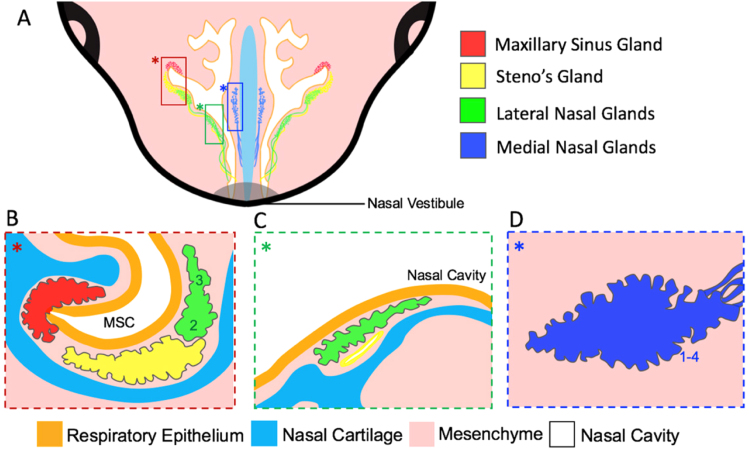
Schematic summary of the morphology and location of the embryonic murine nasal SMGS. (A) Representation of an anterior view of a transverse section of the nasal SMGs surrounding the nasal cavity (white). The SMG ducts of the Steno's gland, Lateral Nasal Glands (LNGs) and Medial Nasal Glands (MNGs) open into the cavity at different locations close to the nasal vestibule, while the maxillary sinus gland (MSG) opens into the maxillary sinus cavity (MSC). Coloured box selections of the distal glands (A) are represented as sagittal sections (B–D). (B) Location of the MSG (red), Steno's gland (yellow) and LNG2 and 3 (green) below the MSC. (C) The LNG5 and an area of the Steno's duct below the respiratory epithelium. (D) MNGs 1–4 in the mesenchyme of the nasal septum. LNGs 6–13 develop adjacent to LNG2, 3 and 5, and undergo gland branching at different locations beneath the nasal cavity along the A/P body axis (not shown).

The SMGs are found in the submucosal connective tissue beneath the respiratory epithelium (RE) of the conductive airways (Fig. 1). The anterior nasal SMGs provide the first line of defence within the airway. The medial and lateral glands are found within the medial and lateral nasal walls respectively, while the sinus glands drain their secretions directly into the sinus cavity (Grüneberg, 1971, May and Tucker, 2015) (Fig. 1). In humans, SMGs are further found within the submucosa between the cartilaginous rings of the distal airways, stretching throughout the trachea and bronchi (Borthwick et al., 1999, Sturgess and Imrie, 1982). In mice, SMGs extend to the anterior trachea, where they are found at high density adjacent to the cricoid cartilage (CC) and develop no further than the sixth cartilaginous tracheal ring (Borthwick et al., 1999, Rawlins and Hogan, 2005).

The SMGs develop through a process of branching morphogenesis. This process, common to other mammalian epithelial organs such as the mammary gland, salivary gland and lung, involves the formation of a single tube from an epithelial sheath that undergoes continual elongation and clefting to create a complex network of branched tubes and terminal buds. Cellular differentiation occurs within these structures to form ductal units within the branches, while end buds differentiate into functional units that transport liquid or gas. In SMGs, these distal functional units are composed of serous and mucous cells that produce airway mucus rich in mucins and bactericidal enzymes (Meyrick et al., 1969, Meyrick and Reid, 1970).

Members of the fibroblast growth factor (FGFs) family of polypeptide proteins have been shown to be involved in branching morphogenesis of other organs such as the lung, salivary and lacrimal glands. The FGF family consists of 22 ligands (FGF1–FGF22) and four cell membrane-bound tyrosine kinase FGF receptors (FGFR1–FGFR4) (Ornitz and Itoh, 2001). The essential requirement for FGF10 and its receptor FGFR2b during lung morphogenesis is emphasised by the shared defects of both *Fgf10* homozygous (−/−) and *Fgfr2b−/−* mice, who die at birth due to agenesis of the lungs (Sekine et al., 1999, Min et al., 1998, De Moerlooze et al., 2000). FGF10 and FGFR2b are also critical for salivary gland duct elongation and branching (Jaskoll et al., 2005, Steinberg et al., 2005). The submandibular salivary gland fails to develop past the initial bud stage at embryonic day E12.5 in both *Fgf10−/−* and *Fgfr2b−/−* mice, and salivary glands are hypoplastic and secrete a reduced volume of saliva in *Fgf10* heterozygous (+/−) adults (May et al., 2015). Mutations in *FGF10* or its receptor *FGFR2b*, lead to Lacrimo Auriculo Dento Digital (LADD) syndrome (OMIM 149730) in humans. This anomaly is characterized by hypoplasia, atresia or aplasia of the salivary glands and the lacrimal glands of the eyes, and obstruction of the nasolacrimal duct (Shiang and Holmes, 1977, Inan et al., 2006). A milder form of this disease, known as Aplasia of Lacrimal and Salivary Glands (ALSG) (OMIM 180920), gives rise to the same symptoms as LADD, most often including xerothalmia (dryness of the eye) and xerostomia (dry mouth) (Wiedemann, 1997; Milunsky et al., 1990).

The Ectodysplasin A (EDA) pathway is also required for morphogenesis of many glandular structures (Mikkola, 2009). A naturally occurring mutation in the *EDA* gene arises in the *Tabby* mouse, leading to defective hair, tooth and salivary gland development (Srivastava et al., 1997). Investigation of the nasal SMGs in the *Tabby* mouse revealed absence of some of the nasal glands, while others, such as Steno's gland, the largest of the nasal glands, also known as the lateral nasal gland 1 (LNG1) (Fig. 1), developed normally in the *Tabby* mouse (Grüneberg 1971). mRNA expression of *Edar*, the receptor for EDA, was found in the tracheal respiratory epithelium during postnatal SMG development (Rawlins and Hogan, 2005). The absence of tracheal SMGs were observed in both the adult *Tabby* mouse and postnatally in the *Edaradd* knockout mouse, which lacks an EDAR signalling adapter molecule, highlighting the requirement of the EDA signalling pathway in successful tracheal SMG morphogenesis (Rawlins and Hogan, 2005). Human patients with hypohidrotic ectodermal dysplasia (HED) have developmental defects in teeth, hair and salivary glands caused by mutations in EDA, EDAR or EDARADD (Mikkola, 2009). Respiratory difficulties and nasal gland defects have also been reported in HED patients with nasal dryness of the nasal mucosa, nasal crusting and abnormal nasal discharge all being symptoms of the disease (Al-Jassim and Swift, 1996, Dietz et al., 2013).

In this study, we have investigated the requirement for FGF10/FGFR2b and EDA signalling during the crucial stages of SMG development among the different nasal gland populations. We conclude that the Steno's gland and sinus glands are predominantly dependent on FGF10/FGFR2b signalling from initial stages of gland budding and elongation, while the medial and lateral glands require this pathway for later gland branching stages. It is suggested that the medial glands are reliant on FGF7/FGFR2b signalling from early development while the lateral glands are more dependent on EDA-mediated signalling. Elucidating these heterogeneous signalling mechanisms will further our understanding in the complex organization and maintenance of these glands, in hopes to combat disease occurrence and progression.

## 2 Material and methods

### 2.1 Experimental animals

*Fgf10*-deficient mice were first generated by Min et al. (1998) (Mouse Genome Informatics ID 1099809). *Fgfr2b−/−* specimens have been previously described (De Moerlooze et al., 2000) (Mouse Genome Informatics ID 2153811). Eda^Ta/Y^ males and Eda^Ta/Ta^ females were used in the analysis of the glands. All procedures and culling methods were performed under a project licence approved by the United Kingdom's Home Office and in accordance with the Animal (Scientific Procedures) Act of 1986, United Kingdom.

### 2.2 Animal collection

For embryo collection, adult mice were mated in the late evening and a midnight mating was assumed. Midday of the day at which a vaginal plug was discovered was recorded as embryonic day (E) 0.5. For postnatal pup collection, the day female mice littered down was recorded as P0. Adult males and females were culled by exposure to rising levels of CO_2_ gas. Primers used to detect wildtype *Fgf10* locus were 5′-GAGGAAATGCTGCGCACAATGTATACTCGG-3′ (Fgf203 forward primer) and 5′-GGATACTGACACATTGTGCCTCAGCCTTTC-3′ (Fgf204 reverse primer) while the mutant *Fgf10* locus was detected by primers 5′-GCTTGGGTGGAGAGGCTATTC-3′ (Fgf233 forward primer) and 5′-CAAGGTGAGATGACAGGAGATC-3′ (Fgf234 reverse primer) of the neo-cassette insert (Sekine et al., 1999). *Fgfr2b* and *Eda* mutants were genotyped as previously described (Charles et al., 2009, De Moerlooze et al., 2000).

### 2.3 Histological Staining and RNA *in situ* hybridisation

Upon collection, embryonic heads were fixed in 4% paraformaldehyde in PBS (PFA) overnight at 4 °C. For tissue collected for *in situ* hybridisation procedures, all solutions used were diethylpyrocarbonate (DEPC) treated. Tissue was dehydrated in increasing methanol concentrations and left overnight at 4 °C in Isopropanol (Sigma Aldrich). Samples were cleared in 1,2,3,4 Tetrahydronapthalene at RT, and embedded in paraffin wax. Alternative serial 9 µm sagittal sections were collected along the left/right axis through the entire embryonic head.

Paraffin embedded sections were dewaxed using Histoclear and rehydrated through an ethanol series. Craniofacial sections were stained using a Trichrome stain of 1% Alcian Blue, Ehrlich's Haematoxylin and 0.5% Sirius Red in saturated Picric Acid.

For *in situ* hybridisation of embryonic nasal tissue with digoxigenin or S35-labelled riboprobes, sections were deparaffinised and rehydrated in decreasing ethanol concentrations in DEPC treated PBS. *In situ* hybridisation technique was carried out using a modified Wilkinson protocol (Wilkinson, 1995). *Fgf7* (Mason et al., 1994), *Fgf10* (Bellusci et al., 1997), *Fgfr2* (Peters et al., 1992) and *Eda* (Tucker et al., 2000) cDNA plasmid vectors and gene inserts used for riboprobe generation have been previously described.

## 3 Results

### 3.1 *Fgf10* and *Fgfr2* are expressed during nasal gland development

**Fig. 2:**
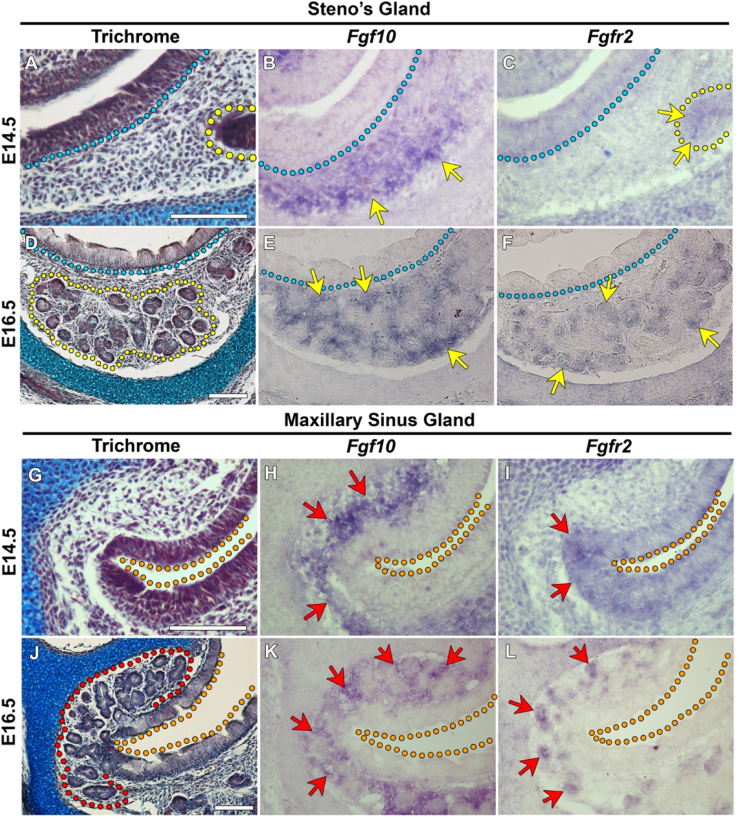
*Fgf10* and *Fgfr2* are expressed during Steno's gland and MSG development. Trichrome stained sagittal sections of the distal Steno's gland (A) and at stage E14.5 of development. (B) At E14.5, *Fgf10* expression is found throughout the mesenchyme beneath the maxillary sinus epithelium in the location of where the Steno's gland will branch, while *Fgfr2* is expressed in the distal epithelial cells of the Steno's duct (C). At E16.5 the Steno's gland is undergoing continual branching (D). *Fgf10* is expressed in the mesenchyme surrounding the distal gland buds (E) and *Fgfr2* is evident in the distal epithelial cells of the Steno's gland end buds (F). (G) Trichrome stained sagittal section of the MSG primordium at E14.5. *Fgf10* is expressed throughout the mesenchyme surrounding the MSG primordium at E14.5 (H) and *Fgfr2* mRNA is detected in the maxillary sinus epithelium, of which the MSG buds (I). By E16.5 when the MSG is branching (J), *Fgf10* is expressed in the mesenchyme surrounding the branching MSG end buds (K). By E16.5, *Fgfr2* expression is lost from the maxillary sinus epithelium but found within the distal end buds of the MSG (L). Yellow line=Steno's gland; blue line=basement membrane of the maxillary sinus epithelium, red line=MSG; orange line=maxillary sinus lumen. Scale bar=100 µm.

To understand the role of the FGF pathway in nasal gland development the expression of *Fgf10* and *Fgfr2* was investigated during embryonic stages of gland initiation and branching morphogenesis. At E14.5, prior to the branching of the distal Steno's gland (Fig. 2A), *Fgf10* expression was observed in the mesenchyme surrounding the extending Steno’s duct (Fig. 2B). *Fgfr2* expression was found within the distal epithelial cells of the elongated Steno's duct at E14.5 (Fig. 2C). During gland branching at E16.5 (Fig. 2D), *Fgf10* expression was apparent throughout the mesenchyme surrounding the end buds of the Steno's gland (Fig. 2E), while *Fgfr2* mRNA was evident within the epithelial cells of the gland buds (Fig. 2F).

*Fgf10* was expressed throughout the mesenchyme adjacent to the maxillary sinus gland (MSG) primordium at E14.5 (Fig. 2G and H), with *Fgfr2* expression observed within the maxillary sinus epithelium from which the MSG buds (Fig. 2I). During MSG branching at E16.5 (Fig. 2J), *Fgf10* expression was maintained throughout the mesenchyme adjacent to the branching MSG end buds (Fig. 2K). At this stage, *Fgfr2* expression was lost within the maxillary sinus epithelium however it was apparent in the distal tips of the branching MSG end buds (Fig. 2L).

At E14.5 the duct of LNG2 had begun its elongation through the middle conchal mesenchyme (Fig. S1A) and the bud of LNG3 had emerged from the epithelium of the middle conchal lip (Fig. S1B) (May and Tucker, 2015). By E16.5, LNG2 and LNG3 were seen branching, close to the maxillary sinus cavity (Fig. S1C). At E14.5, *Fgf10* was expressed in the conchal mesenchyme surrounding both the extending LNG2 (Fig. S1D) and the budding LNG3 (Fig. S1E). At E16.5, *Fgf10* was expressed throughout the mesenchyme surrounding the developing LNG end buds (Fig. S1F). *Fgfr2* was expressed in the ductal epithelial cells of the elongating LNG2 (Fig. S1G) and within the cells of the LNG3 bud (Fig. S1H) at E14.5. As LNG2 and LNG3 were branching at E16.5, *Fgfr2* was expressed within the end bud epithelial cells of LNG2 and LNG3 (Fig. S1I). At E16.5, when the LNG4 duct was elongating beneath the nasal septum, *Fgf10* was also expressed in the mesenchyme surrounding the extending duct with *Fgfr2* within the ductal cells (not shown).

At E14.5, the duct of medial nasal gland (MNG)1 was seen to extend into the mesenchyme of the nasal septum (Fig. S2A) and by E16.5, the MNGs had branched adjacent to the vomeronasal organ (VNO) (Fig. S2B). *Fgf10* was expressed throughout the mesenchyme of the nasal septum at E14.5 when the MNG ducts were elongating (Fig. S2C). At the branching stages of the MNGs at E16.5, *Fgf10* expression surrounded the extending glandular branches (Fig. S2D). *Fgfr2* expression was noted in the epithelial cells of the extending MNG1 duct at E14.5 (Fig. S1E) and *Fgfr2* was expressed within the gland buds at E16.5 (Fig. S1F).

### 3.2 *Fgf10* is critical for Steno's and MSG development

**Fig. 3:**
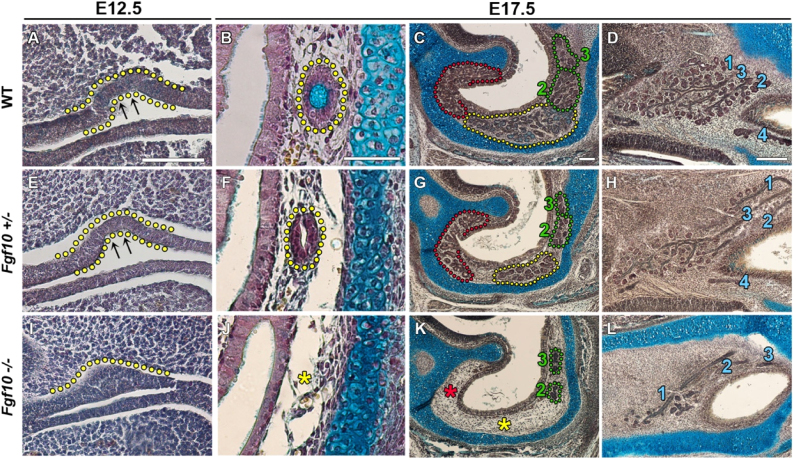
The Steno's gland and MSG fail to develop in *Fgf10−/−* mice. (A) Indentation of the Steno's gland duct is observed in WT animals at E12.5. At E17.5, the Steno's duct (B) and gland (C–D) are well established in WT animals, as well as the MSG (C – red), LNGs (C – green) and MNGs (D). (E) Similar development is seen in *Fgf10*+/− littermates as the Steno's duct dips into the underlying mesenchyme at E12.5. At E17.5, the Steno's duct is found in *Fgf10+/*− animals (F), and the distal gland has developed however a reduction in branching is evident (G – yellow). Reduced branching of the LNGs (G – green), MSG (G – red) and MNGs (H) is also observed in *Fgf10+/−* embryos. (I) No Steno's ductal pit is found in the *Fgf10−/−* mouse. An epithelial placode swelling occurs however, and development appears to arrest at this stage. At E17.5, the Steno's duct (J) and gland (K), and the MSG (K) are completely absent in the *Fgf10−/−* animal. The LNG (K) and MNG (L) ducts extend to their correct location in the *Fgf10−/−* however branching of the distal glands is severely defective. Steno's gland=yellow; MSG=red; LNG2 and 3=green; MNG=blue; asterisks represent absent gland. Column1 scale bar=100 µm, Column2 scale bar=50 µm, Column 3 and 4 scale bar=100 µm.

To define the role of *Fgf10* in anterior nasal gland development, we analysed the gland phenotype in wildtype (WT), *Fgf10+/*− and *Fgf10*−/− mice. At E12.5 the Steno's gland duct arises from the RE and invaginates into the mesenchyme of the middle concha. At this stage an epithelial pit was observed in WT mice (Fig. 3A). By E17.5, the lumenized Steno's duct was observed through a frontal section of the nasal region (Fig. 3B), and the majority of the elongated anterior nasal glands had branched, as evident in a more caudal sagittal section (Fig. 3C). At E17.5, the MNGs had extensively branched throughout the nasal septum mesenchyme (Fig. 3D).

In *Fgf10+/*− mice, the epithelial invagination of the Steno's duct had formed at E12.5, similarly to WT mice (Fig. 3E). At E17.5, the Steno's gland developed in *Fgf10+/−* animals and its duct (Fig. 3F) elongated to the correct location underneath the maxillary sinus (Fig. 3 – yellow), however, the extent of branching was reduced (Fig. 3G). Similarly, branching of the MSG, LNGs, and MNGs was also noticeably reduced in *Fgf10+/−* mice (Fig. 3G and H) compared to that observed in WTs.

In *Fgf10−/−* embryos, no Steno's duct pit was apparent at E12.5 (Fig. 3I). This phenotype indicated that FGF10 signalling is critical for the initial invagination of the duct of the Steno's gland. By E17.5, complete absence of the Steno's duct (Fig. 3J) and gland (Fig. 3K – yellow asterisks) was observed in *Fgf10−/−* mice. Additionally, the MSG was absent with complete loss of *Fgf10* (Fig. 3K – red asterisks). The LNGs were observed in *Fgf10−/−* mice at E17.5 (Fig. 3K). Their ducts budded and elongated to the correct locations when compared to WT specimens, however branching of the distal glands was significantly reduced (Fig. 3K). Furthermore, the MNGs were severely defective with truncated ducts and a considerable reduction in branching, following complete loss of *Fgf10* (Fig. 3L).

### 3.3 *Fgfr2b* is critical for Steno's gland, MSG, LNG4 and MNG development

**Fig. 4:**
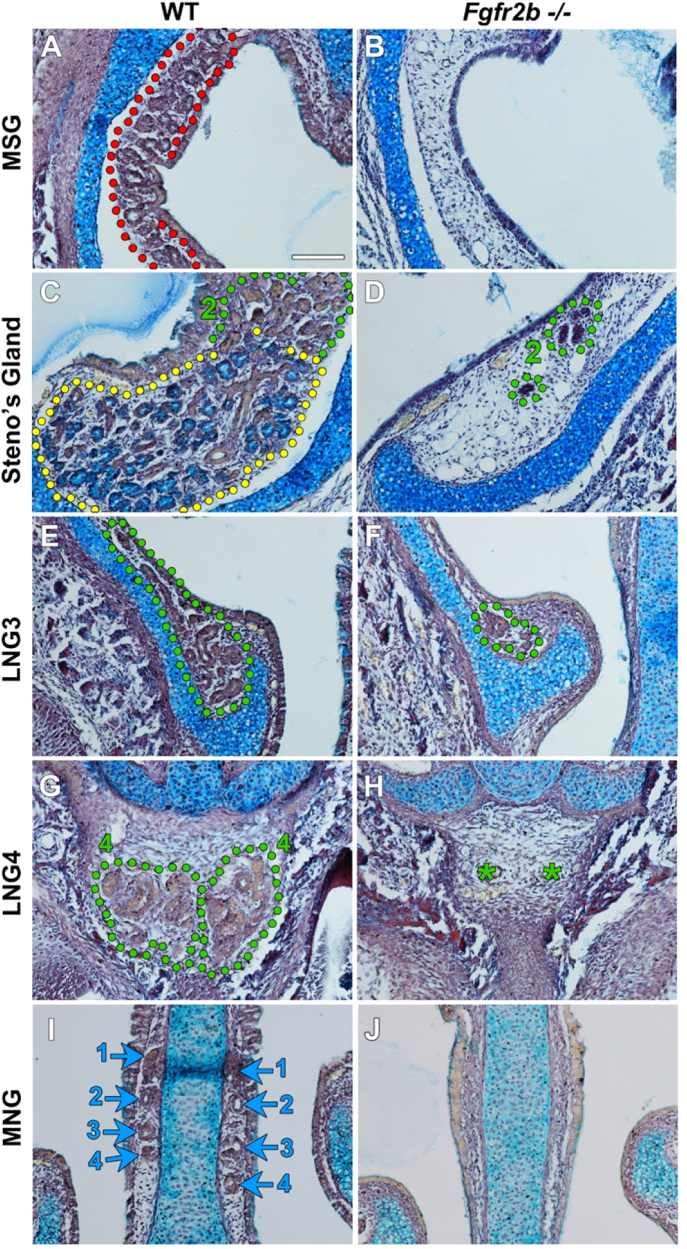
*Fgfr2b* expression is critical for Steno's gland, MSG, LNG 4 and MNG development. (A–B) The MSG develops from the maxillary sinus epithelium in WTs however fails to develop in *Fgfr2b* deficient mice. (C) The distal LNG2 and Steno's gland develop in WT animals. (D) The Steno's gland is completely absent in *Fgfr2b−/−* mice. LNG2 is found in *Fgfr2b−/−* animals however branching of the gland is reduced (D). (E) A branched LNG3 has developed by E18.5 in WT animals. (F) The LNG3 duct extends to the same location in *Fgfr2b−*/*−* mice however branching of the distal gland is reduced. (G–H) Both LNG4 glands are found below the nasal cartilage capsule in WT animals, however are completely absent with the loss of *Fgfr2b.* (I–J) The MNGs are completely absent in *Fgfr2b−/−* mice compared to their WT littermates, indicated by absence of the MNG ducts. Scale bar=100 µm.

Given that only a subset of glands failed to develop in *Fgf10* mutants we decided to assess whether other FGF ligands, working through the same receptor as FGF10, were critical for development of the lateral and medial nasal glands. We therefore investigated the gland phenotype in *Fgfr2b* knockout mice at E18.5, when the majority of glands have extended and started to branch. Similarly to *Fgf10* homozygous mutants, the Steno's gland and MSG were completely absent in *Fgfr2b−*/*−* mice (Fig. 4A–D). The lateral nasal glands (LNG2,3,5) were also present as in the *Fgf10−*/*−*, but again with reduced branching (Fig. 4C–F and data not shown). Unexpectedly, however, LNG4, which normally forms underneath the nasal cartilage capsule, was completely absent in *Fgfr2b* deficient animals at E18.5 (Fig. 4G–H). Additionally, unlike the *Fgf10−/−* phenotype, the MNGs were also completely absent with loss of *Fgfr2b (*Fig. 4I–J). This indicates that other FGFs that bind to FGFR2b are required for MNG and LNG4 duct elongation.

### 3.4 *Fgf7* is expressed during medial nasal gland development

**Fig. 5:**
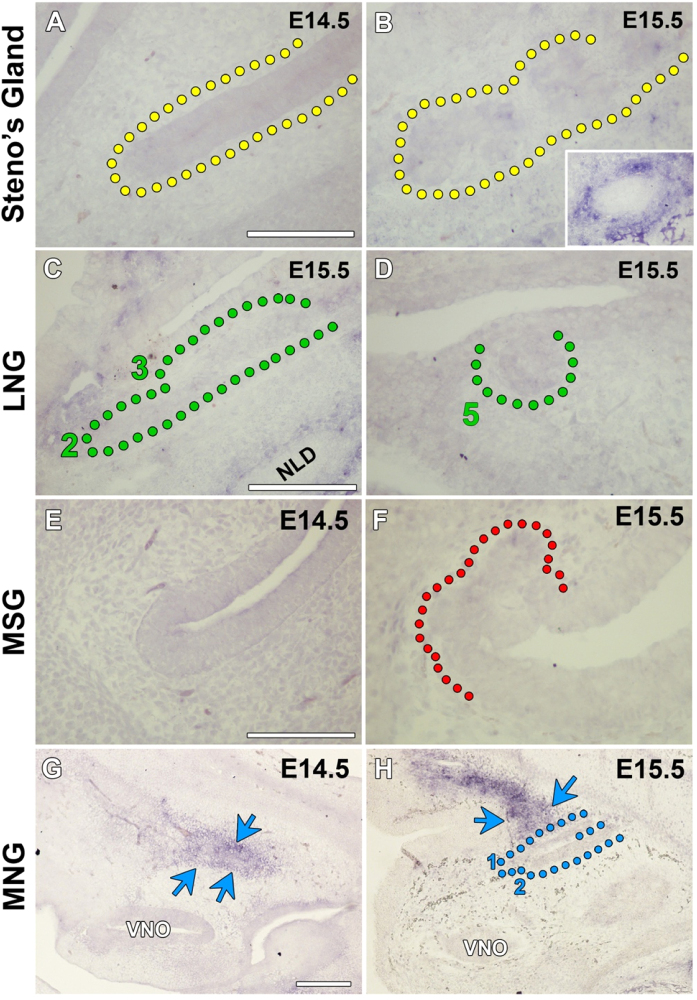
*Fgf7* is expressed in the septal mesenchyme during nasal gland development. (A) At E14.5 *Fgf7* expression is not associated with the elongating Steno's duct. (B) When the Steno's gland is branching at E15.5 no *Fgf7* is observed near the branching gland. *Fgf7* expression is still maintained in the mesenchyme surrounding the NLD (B – inset image). (C) *Fgf7* expression is not found associated with the elongate LNG2 and 3 ducts or (D) the budding LNG5 at E15.5. (E) *Fgf7* expression is not associated with the epithelium where the MSG will bud at E14.5 or (F) surrounding the branching MSG at E15.5. (G) At E14.5, a section through the nasal septum shows *Fgf7* expression throughout the mesenchyme. (H) At E15.5, *Fgf7* expression is seen in the anterior mesenchyme of the nasal septum close to the elongating MNG ducts. A–F scale bar=100 µm, G-H scale bar=200 µm.

Considering the absence of the MNGs in the *Fgfr2b−/*− mouse, yet the presence of MNG ducts and primary branches in the *Fgf10* homozygous embryo, we assessed the expression of *Fgf7* during normal gland development. FGF7 is closely related to FGF10 and also binds to FGFR2b with high affinity (Zhang et al., 2006). *Fgf7* was not found in the mesenchyme surrounding the Steno's gland during any of the duct elongation stages (Fig. 5A) or gland branching (Fig. 5B). *Fgf7* expression was observed surrounding the nasolacrimal duct (NLD) and this was used as a positive control of gene expression (Fig. 5B inset image). No *Fgf7* expression was associated with LNG or MSG development (Fig. 5C–F). *Fgf7* expression was observed throughout the septal mesenchyme at E14.5, close to where the medial nasal glands were forming (Fig. 5G). As the MNG ducts were elongating *Fgf7* expression was maintained in the anterior septal mesenchyme close to the elongating ducts (Fig. 5H). These results suggest that MNG duct elongation may be reliant on FGF7 or another FGF ligand with high affinity to FGFR2 such as FGF1 and FGF22. Multiple FGFs may therefore compensate for the development of these glands.

### 3.5 EDA signalling is essential for LNG and MNG development

**Fig. 6:**
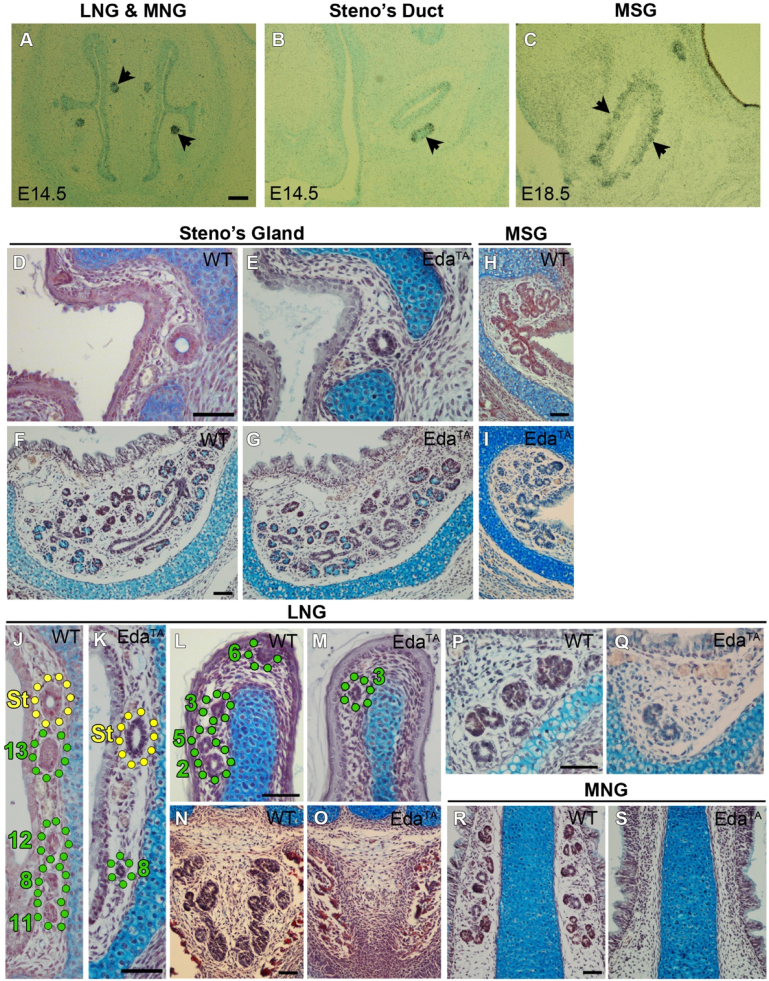
EDA signalling is required for LNG and MNG development. (A–C) *In situ* hybridisation of frontal sections through the nasal chamber during development reveals *Eda* expression in the LNGs and MNGs (A), the Steno's gland (B) and MSGs (C). At E17.5, the Steno's duct (D, F), gland (E, G) and MSGs (H, I) have developed in *Eda*^TA^ mice similar to WT littermates. Ducts of LNG11–13 are absent in the lateral nasal wall of *Eda*^TA^ mice (J–K), and ducts of LNG2, 5 and 6 are absent in the medial conchal lip (L–M). LNG3 and LNG8 ducts develop normally in the *Eda*^TA^ mouse (J–M). Beneath the cartilage of the nasal capsule, LNG4 is absent with the loss of EDA signalling (N–O). Branching of the distal LNG3 gland is reduced in (P–Q) and all of the MNGs are absent (R–S) in the *Eda*^TA^ mouse. Scale bars=100 µm.

Histological analysis has previously indicated that some of the nasal glands are defective in Tabby mutant mice (Grüneberg, 1971). We therefore decided to study the nasal glands of Eda^Ta/Y^ male and Eda^Ta/Ta^ female (Tabby) mice (referred to for simplicity as Eda^Ta^) in more detail to compare to the Fgf mutants. *Edar*, encoding the receptor for EDA, was found to be expressed in the nasal gland epithelium during duct extension and branching (Fig. 6A–C). Despite expressing components of the EDA pathway, the Steno's gland and MSG developed normally in Eda^Ta^ mice (Fig. 6D–I). Investigation of the LNG glands showed that only LNG3 and LNG8 develop in Eda^Ta^ mutants, while no other ducts of the other LNGs were found either in the lateral nasal wall (Fig. 6J–K) or the middle conchal lip (Fig. 6L–M). Similarly, to the *Fgfr2b* knockout mouse, LNG 4, beneath the nasal cartilage capsule, was absent in the Eda^Ta^ mutant (Fig. 6N–O). At a more rostral location, branching of LNG3 was observed to be severely defective in the Eda^Ta^ mouse compared to WT littermates (Fig. 6P–Q). Finally, the MNGs were completely absent in Eda^Ta^ mice (Fig. 6R–S).

## 4 Discussion

### 4.1 *Fgf10* is essential for nasal gland branching morphogenesis

The anterior nasal glands provide a wonderful model for studying branching morphogenesis due to their prolonged duct elongation stage and subsequent gland branching. This allows the investigation of different pathways and signalling molecules involved in these distinct stages of organogenesis. In this study, it was elucidated that *Fgf10* is essential for gland branching of all of the anterior nasal glands. Although branching of the glands occurs in an array of temporal locations, expression of *Fgf10* mRNA was seen throughout the mesenchyme surrounding the gland end buds of all glands during their branching stages. This expression pattern of *Fgf10* is similar to that observed during mammalian lung development where *Fgf10* mRNA is localised to the mesenchyme surrounding newly developing lung buds (Bellusci et al., 1997). In the lung, *Fgf10* has been shown to trigger cell proliferation and act as a chemoattractant (Bellusci et al., 1997, Park et al., 1998), and the expression pattern described in our study suggests a similar role in nasal SMG development. Examination of *Fgf10* mutant mouse also emphasised that with one copy of *Fgf10* present in the heterozygotes, branching of the nasal glands occurs however is reduced. In the complete absence of *Fgf10* in the homozygotes, gland branching is seriously defective with only the presence of severely truncated end buds at the distal ductal tips. The heterozygous phenotype observed is consistent with the lacrimal gland and salivary gland aplasia present in the mouse model and human LADD syndrome (Entesarian et al., 2007, May et al., 2015).

### 4.2 FGF10 plays a heterogeneous role in early anterior nasal gland development

The complete absence of the Steno's gland in the *Fgf10*−/− mouse indicates this growth factor's pivotal role in the development of this structure. This gland differs from the other nasal glands as it buds approximately 24 h earlier and the duct bud invaginates into the mesenchyme as an epithelial indentation, as opposed to a solid swelling of cells (May and Tucker, 2015). In this way the development is more akin to the development of the lung, where the lumen is an integral part of the invaginating epithelium, rather than the lacrimal or salivary glands, where the lumen forms later due to cavitation of a solid cord. The maxillary sinus gland is unusual in that it branches immediately from the maxillary sinus epithelium without formation of an extending duct, so here the loss of the gland is linked to the essential role of *Fgf10* in branching morphogenesis. In contrast to these glands, the lateral nasal glands (LNG), despite expressing *Fgf10* from early stages, had normal duct elongation in the *Fgf10*−/− mice, while the medial nasal glands were also less severely affected. Due to the complexity of biological regulatory networks, removal of one or some of its elements does not necessarily contribute to its overall collapse (Jeong et al., 2000, Albert et al., 2000). Therefore, other signalling molecules appear to compensate for the loss of FGF10.

### 4.3 Other signalling molecules are required for lateral and medial nasal gland development

The loss of the medial nasal glands in the *Fgfr2b*−/− mice indicate that another FGF, working through this receptor, is able to compensate for loss of FGF10. A likely candidate is FGF7. The amino acid core of FGF7 shows over 60% sequence identity with that of *Fgf10*, making it the most structurally similar growth factor to *Fgf10* (Yamasaki et al., 1996). Biological functions are also conserved between these two mesenchymally expressed *Fgfs,* emphasised by both the ligands having high affinity to FGFR2b. During mouse lung development, *Fgf7* is not expressed in the lung mesenchyme during early endoderm branching at E11.5 however *Fgf7* transcripts are detected when the lung is undergoing extensive branching between E13.5 and E14.5 (Bellusci et al., 1997). Early studies using culture of mesenchyme-free endoderm with FGF7 protein showed that it stimulates endoderm stalk extension by inducing cell proliferation (Cardoso et al., 1997). While *Fgf7* expression was not as evident as that of *Fgf10* in the periocular mesenchyme during normal *in vivo* lacrimal gland development, application of an FGF7 bead to lacrimal gland explant cultures induced ectopic gland bud formation, similarly to FGF10 protein application, however not at as high a rate (Makarenkova et al., 2000). In comparison, culture of submandibular salivary glands with FGF7 only gave rise to moderate stalk extension and instead induced epithelial bud enlargement (Koyama et al., 2008). The localisation of *Fgf7* mRNA in the nasal septum mesenchyme from E14.5 to E15.5 when the MNG ducts are budding, elongating and beginning to branch suggest that *Fgf7* function may be utilised by these developing glands in the absence of *Fgf10. Fgf7* mutants do not display gland defects, suggesting that *Fgf10* is able to compensate for loss of *Fgf7* in those glands where both are expressed (Guo et al., 1996).

LNG2, LNG3 and LNG5 all bud and elongate as ducts to the presumptive branching location independent of FGFR2b signalling. Due to the abundant amount of lateral nasal glands found in both rodents and humans, understanding the development of these glands is important in elucidating causes of nasal conditions such as rhinitis and sinusitis. Interestingly the lateral nasal glands, with the exception of LNG3 and 8, failed to form in the *Eda* mutants, while LNG3 had severely reduced branching. These lateral glands therefore rely more heavily on the *EDA* pathway for their development, with the pathway being important for both duct development and branching. The medial nasal glands were also missing, similar to the *Fgfr2b*−/−. These glands therefore require both EDA and FGF signalling for their correct development.

From this study we want to emphasise that nasal SMGS not only adopt contrasting temporal locations and methods of development, they each employ different signalling factors and intracellular cues for their tightly controlled duct elongation and glandular branching. This is important as we can assume defects in different subsets of nasal glands in patients with LADD syndrome and HED. LADD syndrome is caused by mutations in one copy of *Fgfr2b* or *Fgf10*, therefore the heterozygous mice are a good model for this disorder. Importantly branching morphogenesis of all nasal glands was reduced in the *Fgf10*+/*−* mice, suggesting that a similar reduction in branching would be found in the nasal glands of LADD patients. XL-HED is caused by complete loss of *EDA* function, and our results suggest that the lateral and medial nasal glands would be particularly affected in these patients. Such a dramatic loss of these key glands, which cannot be compensated by FGF signalling, would explain the reported defects in this region (Al-Jassim and Swift, 1996, Dietz et al., 2013), although the anterior nasal gland (analogous to the Steno's gland) would be expected to form normally in HED patients.

## Appendix A Supplementary material

Supplementary material Supplementary Figure 1. *Fgf10* and *Fgfr2* are expressed during LNG development. (A-C) Trichrome staining of the LNG2 duct (A) and LNG3 bud (B) at E14.5, LNG2 and LNG3 branching glands at E16.5 (C). (D-E) *Fgf10* expression is found throughout the mesenchyme adjacent to the elongating LNG2 and surround the invaginating LNG3 bud at E14.5. (F) *Fgf10* expression is seen throughout the mesenchyme surrounding the branching LNG2 and LNG3 distal gland buds at E16.5. (G-H) *Fgfr2* is expressed within the epithelial cells of both the LNG2 and LNG3 at E14.5. (I) *Fgfr2* expression is evident within epithelial cells of the branching LNG2 and LNG3 at E16.5. LNGs outlined in green. Scale bar=100 µm. Supplementary Figure 2. *Fgf10* and *Fgfr2* are expressed during MNG development. (A-B) Trichrome staining of the MNG1 duct at E14.5 (A) and MNGs 1–3 at E16.5 (B). VNO=vomeronasal organ. (C-D) A section through the nasal septum showing *Fgf10* expression throughout the mesenchyme adjacent to the elongating MNGs at E14.5 (C) and surrounding the branching MNGs at E16.5 (D). (E–F) *Fgfr2* is expressed in the distal tip of the elongating MNG1 (E) and detected in the distal tips of the branching glandular buds (F). MNG1 duct outlined in blue. Scale bar=200 µm.
