# Supplementary material for: FGF and EDA pathways control initiation and branching of distinct subsets of developing nasal glands
Source: Dev Biol. 2016 Nov 15;419(2):348–56. doi: 10.1016/j.ydbio.2016.08.030 (PMC5145808; doi:10.1016/j.ydbio.2016.08.030)
Supplement: Supplementary file 1 — Supplementary material Supplementary Figure 1. Fgf10 and Fgfr2 are expressed during LNG development. (A-C) Trichrome staining of the LNG2 duct (A) and LNG3 bud (B) at E14.5, LNG2 and LNG3 branching glands at E16.5 (C). (D-E) Fgf10 expression is found throughout the mesenchyme adjacent to the elongating LNG2 and surround the invaginating LNG3 bud at E14.5. (F) Fgf10 expression is seen throughout the mesenchyme surrounding the branching LNG2 and LNG3 distal gland buds at E16.5. (G-H) Fgfr2 is expressed within the epithelial cells of both the LNG2 and LNG3 at E14.5. (I) Fgfr2 expression is evident within epithelial cells of the branching LNG2 and LNG3 at E16.5. LNGs outlined in green. Scale bar=100 µm. Supplementary Figure 2. Fgf10 and Fgfr2 are expressed during MNG development. (A-B) Trichrome staining of the MNG1 duct at E14.5 (A) and MNGs 1–3 at E16.5 (B). VNO=vomeronasal organ. (C-D) A section through the nasal septum showing Fgf10 expression throughout the mesenchyme adjacent to the elongating MNGs at E14.5 (C) and surrounding the branching MNGs at E16.5 (D). (E–F) Fgfr2 is expressed in the distal tip of the elongating MNG1 (E) and detected in the distal tips of the branching glandular buds (F). MNG1 duct outlined in blue. Scale bar=200 µm. [file mmc1.docx]

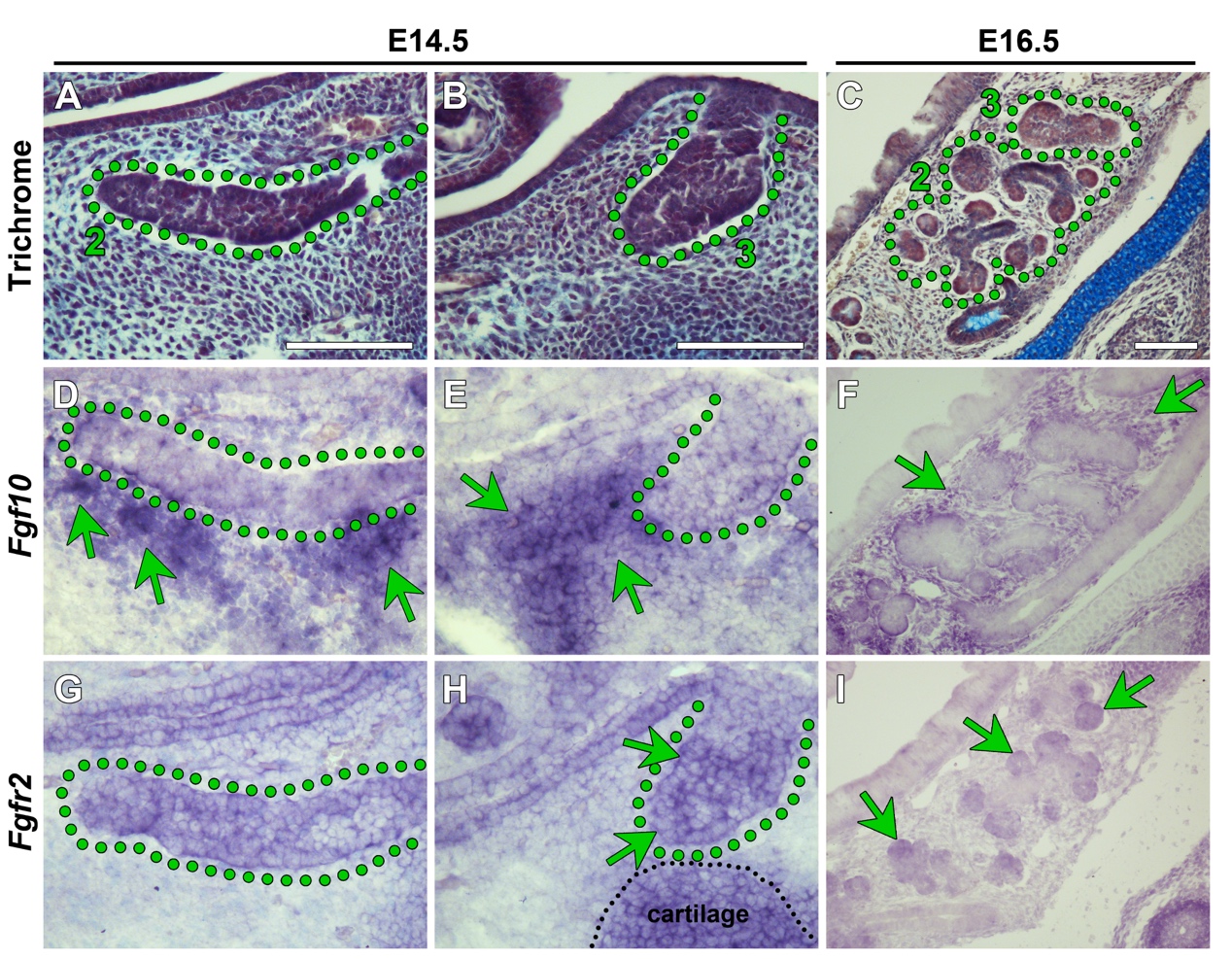


Supplementary Figure 1. *Fgf10* and *Fgfr2* are expressed during LNG development.


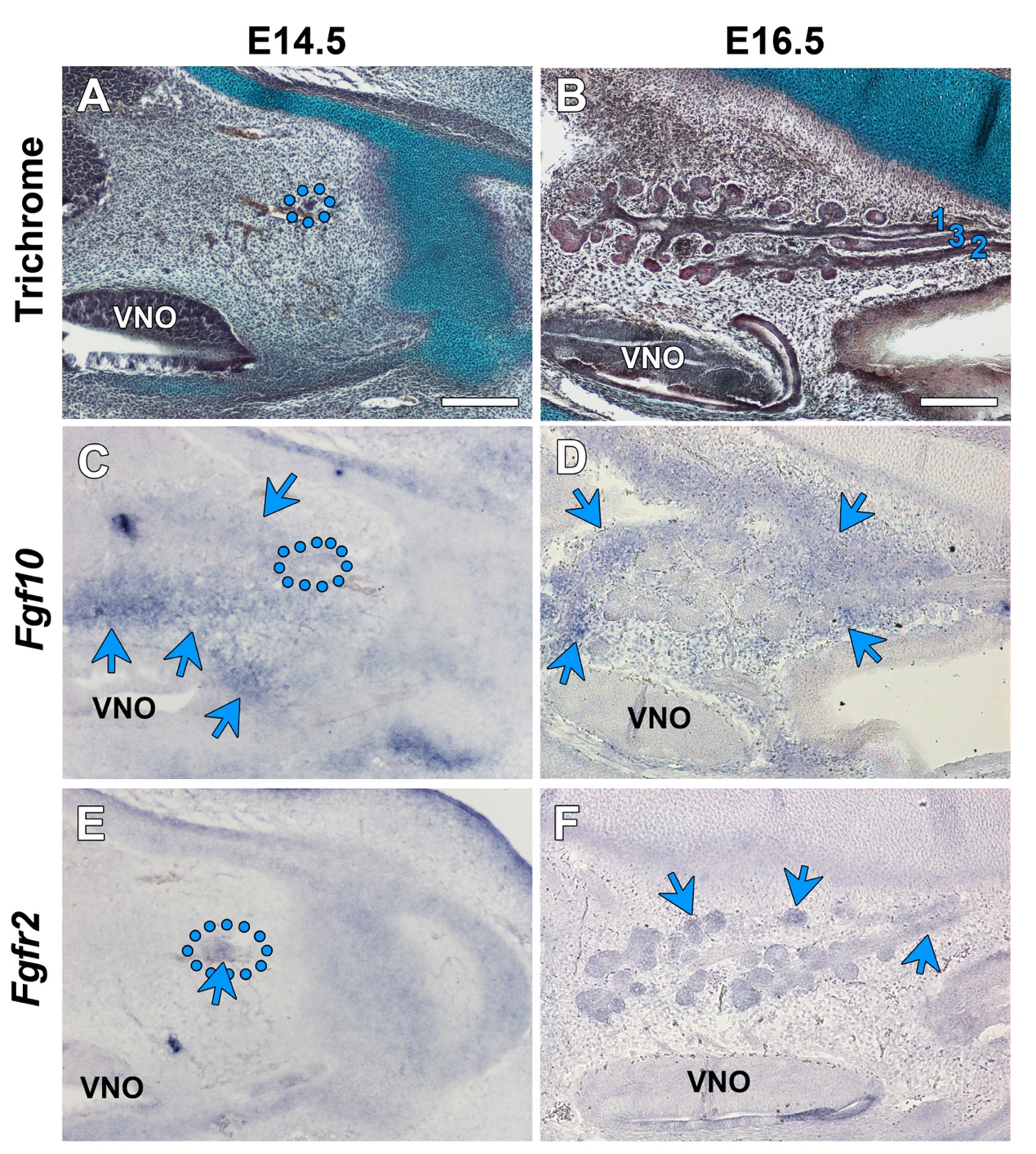


Supplementary Figure 2. *Fgf10* and *Fgfr2* are expressed during MNG development.
